# Supplementary material for: Feasibility, acceptability and initial efficacy of a community-based mental health literacy program delivered by civil society organizations among adults in Kenya: A quasi-experimental study
Source: Glob Ment Health (Camb). 2026 Feb 13;13:e26. doi: 10.1017/gmh.2026.10140 (PMC12914472; doi:10.1017/gmh.2026.10140)
Supplement: Mwangala et al. supplementary material [file S205442512610140Xsup001.docx]

**Supplementary file 1: A comparison of the original *Guide* versus the adapted *Kenyan Guide* and stakeholder suggestions for successful implementation of the adapted *Kenyan Guide***

**A comparison between the original and adapted MHL program**

| **Component** | **Original Guide** | **Adapted Kenyan Guide** |
| --- | --- | --- |
| Language | English | Swahili |
| Modules | Six modules:   1. The stigma of mental illness 2. Understanding mental health and mental illness 3. Information on specific mental illnesses 4. Experiences of mental illness 5. Seeking help and finding support 6. The importance of positive mental health | Nine modules:   1. The stigma of mental health illness 2. The brain and its functions 3. What happens when the brain gets sick 4. Substance abuse and mental health 5. Intimate partner violence and mental health 6. Communication and mental health 7. The importance of positive mental health 8. Resilience and selfcare 9. Seeking help and finding support |
| Mental health illnesses covered | ADHD, Depression, Panic Disorder, Social Anxiety Disorder, PTSD, and obsessive-compulsive disorder; bipolar disorder, eating disorders, schizophrenia, | Common mental health problems e.g. depression, anxiety, PTSD; drug and substance use, and an introduction to the severe disorders e.g. Bipolar, and schizophrenia. |
| Number and duration of sessions | Six lesson sessions, each about 50 minutes. | Nine sessions, each about 1.5 hours. All sessions were covered in 3 days. On a typically day, participants met in the morning (8 am) and ended in the afternoon (1 pm). |
| Mode of delivery | Face-to-face sessions | Face-to-face sessions |
| Place of delivery | School setting | Community spaces including social halls, religious institutions (e.g. churches, mosques) and schools. |
| Client population | School going young people  (13 to 15 years) | Adults in the community (at least 18 years old). |
| Interventionists | Teachers who undergo 1-day training session to become familiar with the Guide and improve their own MHL before implementing it in their classrooms. | Representatives/volunteers of grassroots community-based organizations who underwent 10 days of training on the MHL curriculum. |
| Interventionist trainers | Canadian Mental health specialists including psychiatrists | Kenyan Mental health consultant/psychologist |
| Supervision and mentorship | Mental health practitioners | Global Mental Health practitioner |
| Health system level | school/education system | Community |

**Suggestions from stakeholders for making the implementation of the adapted MHL program a success**

| **Recommendation** | **What was done** |
| --- | --- |
| Using local interventionists for practical reasons e.g. time keeping, understanding of local solutions and for easy sustainability of the program | We mapped out local/grassroots civil society organizations, which were then trained and a few subawarded to implement the program. |
| Minimize excessive client handouts and slides when delivering the content of the program to ensure the message is as simple as possible and to ensure retention of the knowledge shared. | The adapted MHL curriculum incorporated several activities/elements such as brainstorming sessions, roleplays, discussions, experience sharing, case presentations, exercises to simplify the content. |
| Need to have an open and honest communication to establish trust while working with the community | These virtues were emphasized during the training of the MHL program providers and throughout the project implementation. Before implementation, extensive community engagement with local leadership e.g. village elders, chiefs and religious leaders also enhanced this. |
| Flexibility with the 1.5 hours, some sessions will spill over, others will be short. | While an emphasis was placed on being objective during MHL sessions, we encouraged the MHL providers to be open-minded and not overly concerned with having to finish the sessions exactly on the stipulated time. |
| Video about the brain was not clear, and audible. | The video was replaced with a brainstorming and group activity. |
| Have at least 2 facilitators during MHL program implementation. | We trained 3 facilitators from each grassroot organization and at least two had to be present during the implementation. |
